# Identification of a novel HER3 activating mutation homologous to EGFR-L858R in lung cancer

**DOI:** 10.18632/oncotarget.6585

**Published:** 2015-12-09

**Authors:** Ijeoma Umelo, Amir Noeparast, Gang Chen, Marleen Renard, Caroline Geers, Johan Vansteenkiste, Philippe Giron, Olivier De Wever, Erik Teugels, Jacques De Grève

**Affiliations:** ^1^ Laboratory of Molecular Oncology and Department of Medical Oncology, Oncologisch Centrum, UZ Brussel, Vrije Universiteit Brussels, Bruxelles, Belgium; ^2^ Pediatric Hemato-Oncology, UZ Leuven, Leuven, Belgium; ^3^ Department of Pathology, UZ Brussel, Bruxelles, Belgium; ^4^ Department of Pneumology, Universitair Ziekenhuis Leuven, Leuven, Belgium; ^5^ Laboratory of Experimental Cancer Research and Department of Radiotherapy, Universitair Ziekenhuis Gent, Gent, Belgium

**Keywords:** lung cancer, HER3 kinase mutation, HER inhibitor, HER3-V855A

## Abstract

Somatic mutations found within the tyrosine kinase domain (TKD) of the human epidermal growth factor (HER) family of receptors have been implicated in the development and progression of non-small cell lung cancer (NSCLC). However, no conclusive reports have described pathogenic mutations in kinase-impaired HER3. Here, we report a case of an advanced chemotherapy-resistant NSCLC, harboring a novel HER3^V855A^ somatic mutation homologous to the EGFR^L858R^activating mutation. Co-expression of HER3^V855A^ and wild-type HER2 enhances ligand-induced transformation of murine and human cell lines, while HER-targeted inhibitors potently suppress mutant HER3 activity. Consistent with these observations, *in silico* computational modeling predicts that mutant V855A alters the kinase domain and c-terminal end of the HER3 protein. Taken together, these findings provide a basis for the clinical exploration of targeted therapies in *HER3* mutant NSCLC and by extrapolation, in other cancers that more frequently carry somatic *HER3* mutations.

## INTRODUCTION

The identification of activating somatic kinase domain mutations in the human epidermal growth factor (*HER*) or the human erythroblastoma virus B (*ErbB*) family of trans-membrane receptors, which consists of the four homologous members *EGFR* (*HER1*; *ErbB1*), *HER2 (ErbB2), HER3 (ErbB3)* and *HER4 (ErbB4)*, has enabled major advancement in the treatment of non-small cell lung cancer (NSCLC) [1, 2]. These mutations identified in *EGFR, HER2* [2] and *HER4* [3], cluster around their intracellular catalytic tyrosine kinase domain and contribute to disease pathogenesis [2, 4, 5]. EGFR kinase domain mutations present in approximately 5-10% of all NSCLCs [2], highly predict the efficacy of small molecule EGFR or pan-HER tyrosine kinase inhibitors (TKIs) with response rates as high as 70% seen in multiple randomized studies [6-8]. *HER2* driver mutations, on the other hand, are found in less than 2% of NSCLCs [2]. *EGFR* and *HER2* mutation prevalence varies according to patient/tumor selection criteria. Tumor cells that harbor *HER2* mutations exhibit preclinical and clinical sensitivity to the pan-HER inhibitor afatinib [9-11]. The few lung cancer-derived *HER4* kinase mutations reported to date have not been extensively studied. *HER4* has an attenuating role in *HER* signaling and mutations rather create a loss of function [12, 13]. While *HER3* mutations have been reported in some human cancers [14, 15], no conclusive reports to date have described *HER3*-related pathogenic mutations in lung cancer [16-18].

*HER3* is unique among the *HER* receptor family members as it is generally considered to lack or have impaired tyrosine kinase activity due to the absence of critical amino acid residues within its kinase domain securing it in an inactive conformation [18-21]. Despite this perceived absence of intrinsic tyrosine kinase activation, *HER3* plays a critical role in the signaling of the other HER members. Unlike other *HER* receptors, *HER3* does not form stable ligand-induced homodimers [22], but upon ligand binding acts as an allosteric activator of its other HER partners, particularly HER2. This activation results in the propagation of a potent signaling cascade [18, 20, 23] and can also play a role in carcinogenesis [24, 25]. In addition, *HER3* contains six binding sites for the p85 regulatory subunit of phosphoinositide 3-kinase (PI3K) that are not present in *EGFR* or *HER2*, establishing *HER3* as a strong intermediary for PI3K/AKT signaling [18, 26].

Here, we report on a novel V855A mutation located in exon 21 of the *HER3* tyrosine kinase domain and found in the tumor specimen of an adolescent patient with a chemotherapy-resistant advanced NSCLC. Interestingly, this mutation maps at a position homologous to the prevalent EGFR-L858R driver mutation [27] and we thus hypothesized that this mutant HER3 may have a functional impact. We demonstrate that HER3-V855A alters HER3 protein structure and confers a gain-of-function phenotype when co-expressed with HER2 but not with EGFR. We also demonstrate that HER-specific therapeutics can effectively suppress mutant V855A transforming potential. These preclinical results provide a rationale for the clinical exploration of anti-HER therapies in *HER3* mutant lung cancer and by extrapolation in other cancers that more frequently harbor *HER3* somatic mutations.

## RESULTS

### Clinical presentation

**Figure 1:**
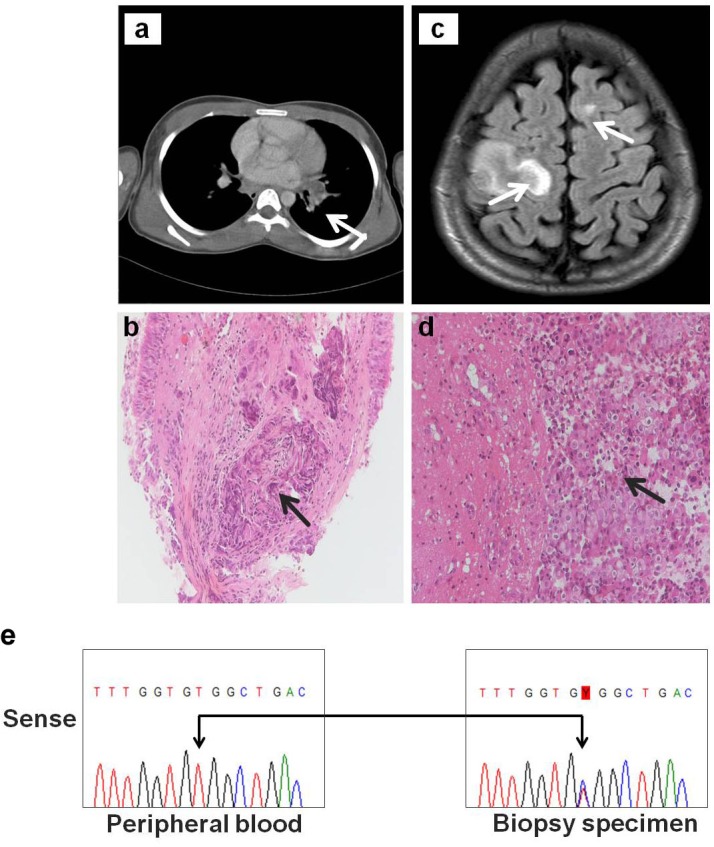
A novel HER3 somatic mutation in NSCLC Clinical findings of a metastatic lung adenocarcinoma *in a 14 year old male:* **a**. axial computed tomography (CT) image showing primary lesion in left bronchus of patient (arrow), **b**. H&E stained section of transbronchial biopsy specimen showing nests of poorly differentiated adenocarcinoma (arrow) infiltrating the bronchial mucosa (original magnification, x20), **c**. MRI scan of patient showing metastatic brain lesions in the white matter of both the left and right frontal lobes (arrows), **d**. H&E stained section showing metastatic lung adenocarcinoma in brain biopsy. Glial tissue is invaded by nests and sheets of tumor cells, growing in a cohesive pattern (arrow) (original magnification, x20) and **e**. Sanger sequencing chromatograms covering exon 21 of the HER3 gene revealing a double peak with a novel T-to-C base pair change resulting in a V855A mutation (arrows) in the pleural lung biopsy specimen of the patient (right panel). Patient's peripheral blood specimen (left panel) reveal the wild-type HER3 sequence only.

A 14-year-old Caucasian male presented for evaluation with a paresis affecting his left arm. A brain MRI demonstrated diffuse multiple lesions with uptake of contrast and surrounding edema (Fig. 1c) while a subsequent brain biopsy revealed the presence of metastasis of an adenocarcinoma (Fig. 1d). The immunohistochemical profile of the tumor (CK7 and TTF1 positive) suggested a primary origin from the lung. Further screening via computed tomography (CT) revealed the presence of a primary lesion in the left bronchus (Fig. 1a). The screening also demonstrated severe metastatic spread with multiple thoracic and abdominal adenopathies and metastases in the liver and kidneys (data not shown). A transbronchial biopsy confirmed the presence of a poorly differentiated adenocarcinoma infiltrating the normal bronchial tissue, with roughly 40% of the specimen consisting of tumor cells (Fig. 1b).

### Identification of a novel HER3-V855A mutation

A single arm multicenter phase II clinical study initiated in 2006 (FIELT1 study; NCT00339586) was coordinated by our department to evaluate the safety and efficacy of first-line erlotinib in patients with advanced NSCLC with a documented *EGFR* kinase mutation. Patients were pre-selected on the basis of 2 criteria: adenocarcinoma with little or no smoking history. Patients with an *EGFR* mutation in their tumor were then treated with erlotinib (150mg/day) until progression [28]. DNA isolated from formalin-fixed lung cancer biopsy samples, including the specimen from the 14 year old male patient, were screened for mutations in the kinase domain (exons 18 through 21) of all four *HER* family genes by the denaturing gradient gel electrophoresis (DGGE) method that detects as low as 5% mutant species in a wild-type background [29]. Additional screening was also performed on exons previously reported to harbor hotspot mutations in *KRAS* and *BRAF* [2]. From a total of 210 screened samples, eighteen previously reported *EGFR* pathogenic mutations (n=55) and two (n=5) previously reported *HER2* insertion mutations were identified [29]. As depicted in Fig. 1e, further examination of DNA extracted from the lung cancer specimen of the 14-year-old case-study revealed a double peak at nucleotide c.2564 located in exon 21 of the *HER3* gene (NM_001982). This indicated the presence of a mutated allele with a T-to-C base-pair substitution, predicted to substitute valine (GTG) to alanine (GCG) at codon 855 (p. Val855Ala; NP_001973) of the HER3 activation loop. The HER3-V855A mutation was detected in the tumor sample, but was not found in the patient's peripheral blood DNA (Fig. 1e), confirming that the mutation was of somatic origin. Additional genomic analysis of the patient's lung tumor specimen did not reveal any additional mutations in the other tested genes. The disease progressed despite treatment with VIDE (vincristin, ifosfamide, doxorubicin and etoposide) although there was an initial objective response. Unfortunately, demise occurred before the patient could be included by amendment in an exploratory lung cancer phase II study with afatinib [11].

### Homology between the HER3-V855A and EGFR-L858R kinase mutation

*EGFR* pathogenic mutations sensitize in varying degrees to inhibition by small molecule TKIs [27]. These mutations include both class I short in-frame deletions and class II missense mutations. One of these mutations, the L858R(Leucine → Arginine) missense mutation occurs at a highly conserved amino acid among protein kinases and is found in exon 21 of the EGFR kinase domain [30]. In addition, this single nucleotide substitution has the highest prevalence of any activating *EGFR* kinase domain missense mutation, accounting for approximately 41% of *EGFR* kinase mutations [31]. Moreover, EGFR-L858R leads to increased sensitivity to EGFR TKIs, albeit with less dramatic response than the exon 19 deletion mutation [27].

**Figure 2:**
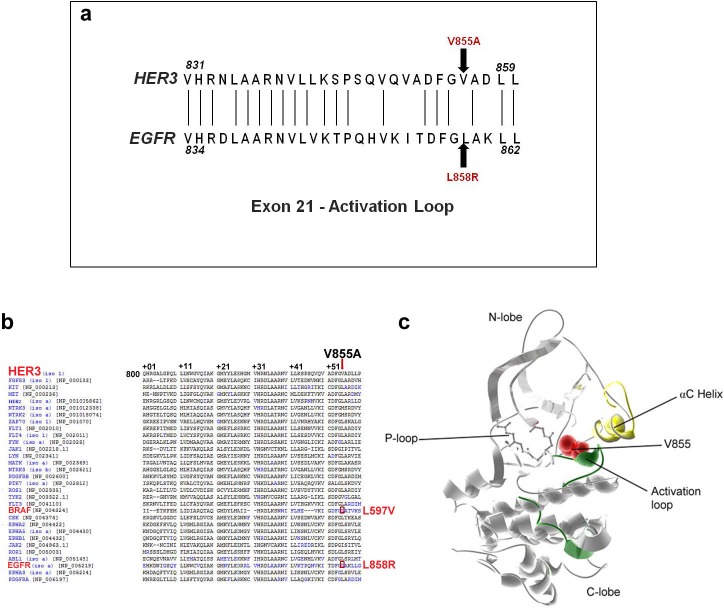
Protein structure visualization of the novel HER3-V855A mutation **a**. Partial amino acid alignment of exon 21 sequence of the HER3 and EGFR tyrosine kinase domain. Also shown are the positions affected by the EGFR-L858R mutation and the novel HER3-V855A mutation (arrows). **b**. Partial amino acid alignment of the kinase domain of HER3 with other receptor tyrosine kinases adapted from the mutagrator kinase mutation interpretation tool reveals mutations (blue) at analogous residues. The HER3-V855A mutation is also analogous to the BRAF-L597V kinase mutation (red box). **c**. 3D structure of HER3 kinase domain (PDB: 3LMG) depicting the location of V855A mutation (red van der Waals radii).

To analyze the location and significance of the novel HER3-V855A mutation, we performed protein sequence alignment of exon 21 of the EGFR and HER3. Although, the amino acid at position 855 in HER3 is not conserved relative to EGFR, the mutated amino acid remarkably maps at a position analogous to the location of the EGFR-L858R mutation (Fig. 2a). Further analysis with the Mutagrator kinase mutation interpretation tool [32] reveals that the mutated V855 residue also has positional homology to the lung cancer-derived BRAF- L597V kinase mutation [33] (Fig. 2b). BRAF-L597V is classified as an intermediate kinase active variant (approximately100 fold elevated BRAF activity compared to wild-type) that modestly increases ERK activation [34]. The mutated residue is also highly conserved across HER3 homologs among different mammalian species (data not shown), which further indicates that the V855A mutation may have a functional effect. In addition, analysis of the crystal structure of the HER3 kinase domain depicting the mapped location of the mutated residue, demonstrates its functional relevance. The V855 residue is part of a conserved sequence motif (also includes L858 and L859) which stabilizes the inactive position of the αC helix [20](Fig. 2c), and we propose that the amino acid substitution may likely affect protein kinase activity.

### HER3^V855A^ expressed with HER2^WT^ enhances neuregulin1β-induced transformation in a null cellular model

**Figure 3:**
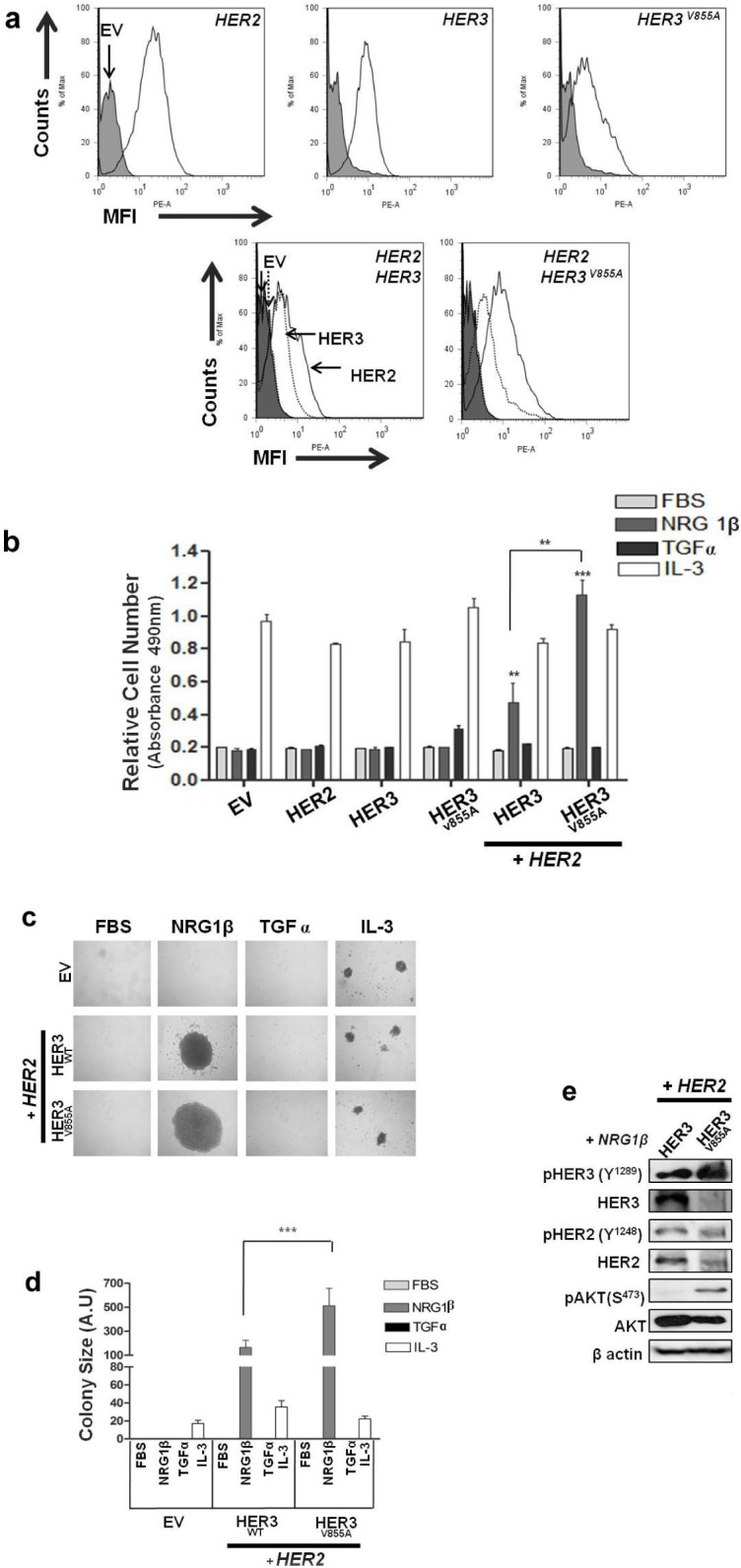
HER3-V855A combined with HER2 enhances neuregulin 1β-induced activity in transformed Ba/F3 cells **a**. Selected Ba/F3 transfectants were analyzed for cell surface protein expression by staining with HER-specific antibodies to confirm recombinant protein expression. HER2 and HER3 co-transfectants were labeled with PE-conjugated anti-HER2 or PE-conjugated anti-HER3 antibodies. **b**. Ba/F3 transfectants were cultured for 7 days in the absence or presence of the indicated stimulants. Cell growth was analyzed by the MTS assay. **c**. Ba/F3 co-transfectants were subjected to a methyl cellulose based colony formation assay in the presence of the indicated stimulants for 21 days. Magnification, 20X. **d**. Colonies were quantified using computerized photoshop CS6 analysis. **e**. Ba/F3 co-transfectants were cultured in the presence of NRGβ for 5 days. Total cell lysates were analyzed by immunoblot analysis using the indicated antibodies. EV, empty vector. Also see supplemental Figure 1 and 2.

*HER3* has been described as a contributor to oncogenic transformation and tumorigenesis, particularly when combined with its *HER2* dimerization partner [19]. Therefore, we hypothesized that the HER3 kinase mutation may cause a pathogenic phenotype when co-expressed with HER2 in a cellular system. To this end, we used the Ba/F3 model system to determine the functional impact of HER3-V855A. Ba/F3 cells are dependent on interleukin-3 (IL-3) for mitogenesis and lack endogenous expression of all the HER receptors (although very low *HER3* expression has been detected in reverse-transcriptase polymerase chain reaction (RT-PCR) studies [35]), enabling us to focus on the properties of the mutant *HER3* in a basically null background. Ba/F3 cells were co-transfected with vectors encoding differing forms of the *HER* receptors (wild-type *HER2*, wild-type *HER3* or mutant *HER3*). Stable cell populations were screened by fluorescence-activated cell sorting (FACS) and selected based on matched expression level (within two fold levels) of their relevant HER at the cell surface (Fig. 3a).

To determine the transforming potential of HER3-V855A in the context of IL-3 -independent growth, Ba/F3 transfectants were grown in the absence or presence of IL-3, or HER cognate ligands (neuregulin1β (NRG1β) or transforming growth factor α (TGFα)). NRG1β is a key activator of HER3 and its expression by NSCLC cells has been described to promote autocrine activation of the HER2/HER3 complex [36]. In contrast, TGFα activates EGFR and is highly expressed in NSCLC [37]. As shown in Fig. 3b, Ba/F3 cells singly expressing wild-type HER3 or mutant HER3-V855A were unable to grow in IL-3 free culture conditions, even when supplemented with NRG1β or TGFα ligands. On the other hand, while still no growth response was observed when co-expressing each HER3 construct with wild-type HER2 in absence of IL-3, a significantly increased growth response upon NRG1β stimulation was observed with the mutant HER3 combination compared to the wild-type combination (p < 0.01). The extent of NRG1β-induced growth in the HER3-V855A/HER2 co-transfectant was even greater than levels observed in IL-3 treatment conditions (Fig. 3b). Furthermore, treatment with TGFα did not induce a growth response in both wild-type and mutant HER3 co-transfectants (Fig. 3b), further confirming that the presence of a HER3-specific cognate ligand is required to promote HER3/HER2 biological activity.

To assess the ability of HER3-V855A to form colonies we performed a methyl cellulose-based colony formation assay. As shown in Fig 3c & 3d, while NRG1β treatment did not induce an increase in colony number between the wild-type and mutant HER3 co-transfectants, colony size was significantly greater inHER3-V855A compared to wild-type HER3 (p < 0.001). The colony size upon NRG1β treatment was approximately 25-fold greater than under IL-3 treatment with the mutant HER3 co-transfectant (Fig. 3d).

We also investigated the functional relevance of stable Ba/F3 transfectants co-expressing HER3-V855A and EGFR (Supplemental Fig. 1a). While Ba/F3 cells co-expressing HER3-V855A and EGFR exerted a robust growth response to TGFα treatment (Supplemental Fig. 1b), they were unable to form colonies with the same treatment (Supplemental Fig. 1c). In contrast, Ba/F3 cells co-expressing wild-type HER3 and EGFR not only formed colonies in the presence of TGFα (Supplemental Fig. 1c), but also demonstrated equal TGFα-induced growth response compared to the mutant HER3-V855A combination (Supplemental Fig. 1b). These data suggest that the pathogenic effects associated with the HER3-V855A mutation is not induced by heterodimerization with EGFR (upon TGFα stimulation).

We next examined the effect of chronic treatment with NRG1β on HER3/HER2 phosphorylation and their downstream targets AKT and ERK 1/2 in the Ba/F3 co-transfectants. As shown in Figure 3e, a five-day chronic treatment with NRG1β specifically increased levels of phosphorylated HER3 and AKT in HER3-V855A compared to wild-type HER3. In contrast, while chronic NRG1β treatment induced HER2 and ERK1/2 phosphorylation in HER3-V855A, their levels were not enhanced compared to wild-type. Of note, chronic treatment with NRG1β neither induced HER3 or HER2 phosphorylation in Ba/F3 cells expressing HER3-V855A combined with a kinase-dead (KD) HER2 isoform (Supplemental Fig. 2b). This finding further reveals that a competent HER2 receptor is required for mutant V855A transforming activity.

### HER3^V855A^ enhances neuregulin 1β-induced trans-phosphorylation of HER2^WT^

**Figure 4:**
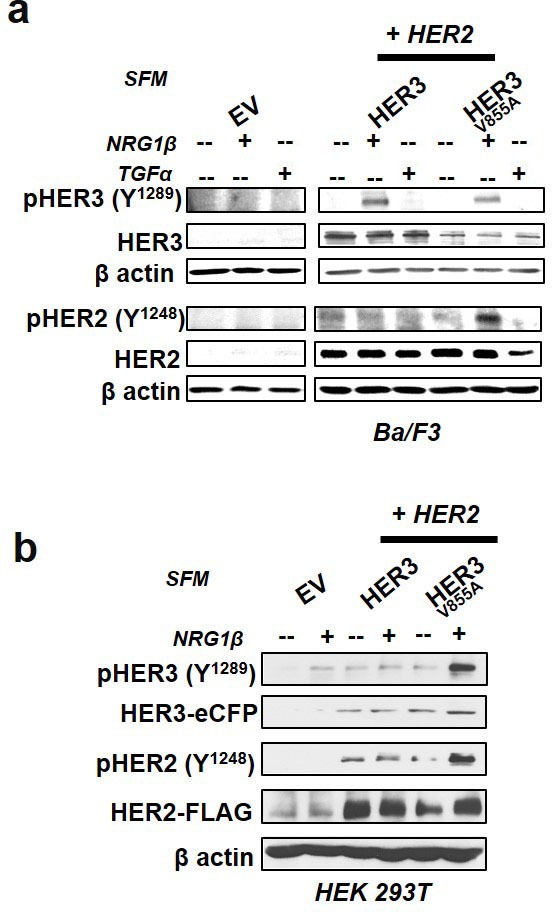
HER3-V855A enhances neuregulin 1β-induced activation of HER2 in transformed cells **a**., **b**. Western blot analysis of Ba/F3 and HEK 293T transfectants treated for 16hrs in serum free conditions (SFM) and stimulated for 10 minutes with the indicated EGFR/HER cognate ligands. The cells were subsequently lysed and subjected to immunoblot analyses. Also see supplemental Figure 3.

Tyrosine trans-phosphorylation is a major event in HER signaling [38]. To examine if HER3-V855A enhances trans-phosphorylation of HER2, we performed immunoblot analysis on Ba/F3 and HEK 293Tlysates after 16hr incubation in serum-free conditions followed by10 minute acute ligand stimulation. HER3-V855A enhanced trans-phosphorylation of HER2 at Y1248 in both Ba/F3 and HEK 293T cells after NRG1β stimulation (Fig. 4a and 4b).

In addition, while acute treatment with NRG1β was essential for the induction of HER3 phosphorylation in wild-type and mutant HER3 co-transfectants, HER3 phosphorylation was absent in similar treatment conditions with corresponding single transfectants (Fig. 4a and supplemental Fig. 3a). NRG1β-induced phosphorylation of mutant HER3 was strongly enhanced in HEK 293T cells, whereas the observed effect in Ba/F3 transfectants was more modest (Fig. 4a and 4b). Likewise, a modest increase in ligand-induced phosphorylation of HER2 and HER3 was observed in transformed H292 cells (Supplemental Fig 3b). H292 cells are NSCLC cells that display similar mucoepidermoid features as normal lung (non-cancerous) cells. Consistent with previous functional experiments (see Fig. 3), TGFα was unable to induce HER phosphorylation in transformed Ba/F3 cells (Fig. 4a).

### Enhanced interaction of HER2 with HER3^V855A^

**Figure 5:**
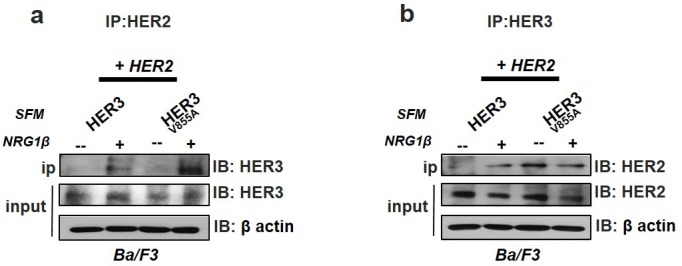
Enhanced interaction of HER2 with HER3-V855A Ba/F3 transfectants co-expressing HER2 and HER3 were treated in serum free conditions (SFM) for 1 hr followed by a 10-minute stimulation with NRG1β Total cell lysates were co-immunoprecipitated with either HER2 or HER3 antibodies, followed by western blot analysis. 20% input levels are indicated.

To further confirm that the V855A mutation provides increased activity to HER3 through enhanced physical interaction with HER2, we performed co-immunoprecipitaton experiments on Ba/F3 co-transfectants stimulated with or without NRG1β (10 minutes) after 1 hr serum starvation. Pull down of HER3 on HER2 immunoprecipitates revealed enhanced interaction of HER2 with HER3-V855A relative to wild-type HER3, specifically after incubation with NRG1β (Fig 5a). Interestingly, reciprocal analysis with HER3 immunoprecipitates demonstrates that HER2 already interacts with the mutant HER3 complex in the absence of NRG1β (with no observed effects with ligand stimulation), while NRG1β stimulation is needed for interaction with the wild-type HER3 protein (Fig. 5b). Of note, the cell surface protein expression of HER2 is much higher than HER3 in the transfected Ba/F3 cells (Fig. 3a). This thus infers that a smaller fraction of the HER3 complex is pulled down in Fig. 5b, and may also result in differences in the amount of proteins stimulated by NRG1β.

### Efficacy of HER inhibitors on transforming activity of HER3^V855A^

**Figure 6:**
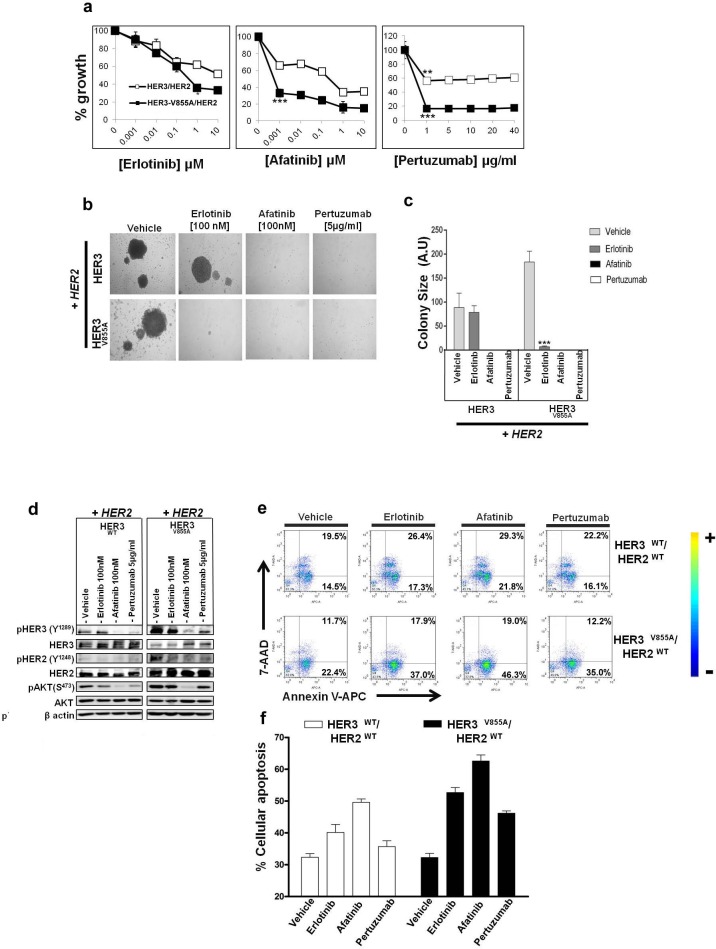
Effectiveness of HER inhibitors on biological activity of HER3-V855A in transformed Ba/F3 cells **a**. Dose-dependent growth inhibition of Ba/F3 co-transfectants treated with the indicated inhibitors and grown in the presence of NRG1β for 7 days. Cell growth was assessed by the MTS assay. **b**. Ba/F3 co-transfectants were subjected to a methyl cellulose based colony formation assay in the presence of NRG1β and treated with the indicated inhibitors or vehicle control (0.01% DMSO) for 21 days. **c**. Colonies were quantified using computerized photoshop CS6 analysis. Magnification, 20X (Mann Whitney U test: ***,p< 0.001. **d**. Following overnight serum starvation, Ba/F3 co-transfectants were treated with the indicated inhibitors or vehicle control (0.01% DMSO) for 2 hours and then stimulated with 100ng/mL of NRG1β for 10 minutes. Immunoblot analysis was performed with the indicated antibodies. **e**. Ba/F3 co-transfectants treated with the indicated inhibitors (erlotinib (1μM), afatinib (1μM) and pertuzumab (20 μg/mL)) or vehicle control (0.1% DMSO) were grown in the presence of NRG1β for 5 days and evaluated by the Annexin V-APC/7-AADbinding assay. **f**. Quantification of apoptosis with the sum of early and late apoptotic cell populations (only Annexin V stained) in the y-axis plot. Results are the averages from two replicate studies. Also see Table 1.

To investigate whether HER3-V855A can be therapeutically targeted; we examined the growth inhibitory effects of inhibitors targeting the extracellular and kinase domain of the HER receptors. These inhibitors include: erlotinib, an FDA approved reversible TKI indicated for the treatment of advanced NSCLC and pancreatic cancer, afatinib, a second generation irreversible ErbB family blocker also approved for the treatment of advanced NSCLC and pertuzumab, a monoclonal antibody that targets the extracellular region of HER2 preventing it from dimerizing with its other HER partners and approved for the treatment of HER2 amplified breast cancer in combination with trastuzumab [39-41]. To assess the inhibitory effect of these molecules on cell growth, Ba/F3 transfectants growing in NRG1β containing medium were treated with increasing concentrations of these inhibitors for a period of seven days. Afatinib and pertuzumab each demonstrated high efficacy in inhibiting the growth of Ba/F3 cells co-expressing HER3-V855A and wild-type HER2 (Fig. 6a), with IC_50_ values of < 1 nmol/L and < 1μg/mL respectively (Table 1). Erlotinib, on the other hand, had a much weaker inhibitory effect on the mutant V855A combination (Fig. 6a) with a calculated IC_50_ value of 344±4nmol/L (Table 1). Ba/F3 cells co-expressing wild-type forms of HER3 and HER2 were highly resistant to erlotinib treatment (7000±106 nmol/L), but displayed sensitivity to afatinib with an IC_50_ value of 208 ± 39nmol/L, two logs less sensitive than the mutant form (Table 1).

**Table 1:** IC_50_ values for growth inhibition by HER inhibitors assessed by the MTS colorimetric assay

Ba/F3 transfectant	HER3 wild type HER2 wild type	HER3 V855A HER2 wild type
**ERIotinib** **IC_50_ μM**	Resistant	0.3±0.006
**Afatinib** **IC_50_ μM**	0.2±0.004	<0.001
**Pertuzumab** **IC_50_ μM**	Resistant	<1

**Cell growth was measured following a 7 day drug treatment in the presence of NRG1β.** IC_50_ values were calculated by the BioSoft ^®^ CalcuSyn 2.0 Software

To assess the effect of the inhibitors on colony formation, Ba/F3 co-transfectants were seeded onto methyl-cellulose and treated with HER inhibitors in the presence of NRG1β. As shown in Fig 6b, afatinib (100 nmol/L) and pertuzumab (5μg/mL) were effective in suppressing the formation of colonies in wild-type HER3 and mutant V855A co-transfectants. As expected, erlotinib (100nmol/L) was less effective than the other HER inhibitors in suppressing colony formation. The HER3-V855A co-transfectantformed significantly smaller colonies (7.2 ± 1.4) compared to the wild-type HER3 combination (78.9 ± 13.7) under erlotinib treatment (Fig. 5b and 5c).

We further examined the effects of the inhibitors on HER-related signaling activity and survival using the Ba/F3 model system. Afatinib (100nmol/L) potently inhibited NRG1β-induced phosphorylation of HER3, wild type HER2 and AKT (Fig. 6d). Pertuzumab (5 μg/mL) treatment induced less potent effects compared to afatinib, with significant reduction seen in mutant HER3 and wild-type HER2 phosphorylation, but not in active levels of AKT (Fig. 5d). Treatment with erlotinib (100nmol/L) also inhibited the HER pathway, albeit less potently than afatinib or pertuzumab treatment (Fig. 6d). As determined by the annexin-V/7-AAD binding assay, treatment with afatinib in the presence of NRG1β induced a two-fold higher fraction of apoptotic cells compared to vehicle conditions (30.3 ± 0.6%). Erlotinib and pertuzumab were less effective, yielding respectively a 20.4 ± 0.29% and 13.9±0.6% increase in apoptotic cells compared to vehicle (Fig. 5e). All tested inhibitors were also less effective in suppressing HER-related activity and survival in the wild-type HER3/HER2 co-transfectant (Fig. 5d and 5e), indicating that tumors harboring HER3-V855A may predict response to targeted therapy.

### Impact of V855A on HER3 protein structure

**Figure 7:**
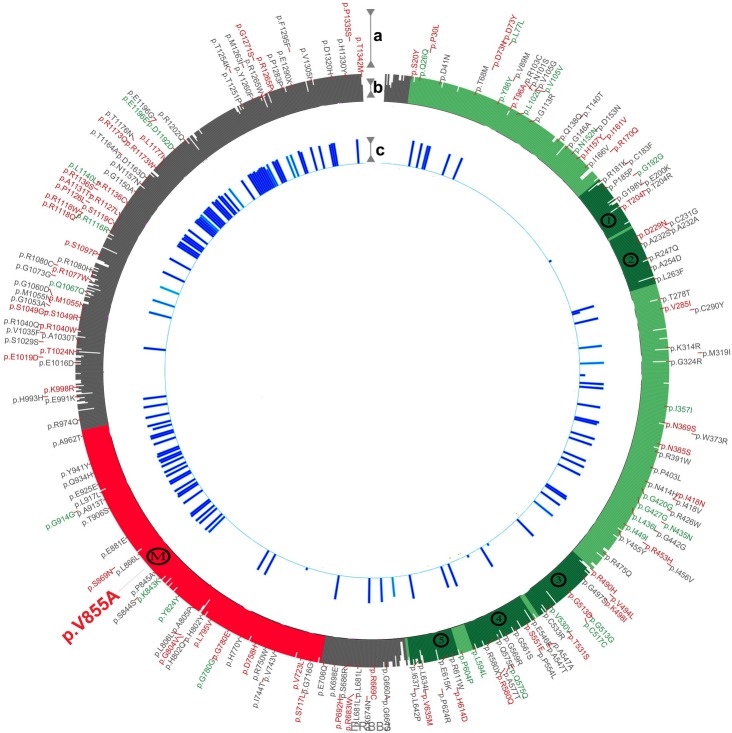
Prediction track depicting the impact of V855A on HER3 protein structure **a**. SNPs from dbSNP (http://www.ncbi.nlm.nih.gov/SNP) were plotted with synonymous, non-synonymous and non-validated SNPs in green, red and gray respectively. **b**. Alignment generated conservation track shows the extracellular region (residues 20-643) in light green, the kinase domain (709-966) in red and the regulatory carboxyl-terminal end (967 - 1342) in gray. The cysteine rich furine-like repeats have been marked with numbers (1-5) along the track. The position of the valine to alanine substitution has been marked with an M. **c**. Server predicted wild-type and mutated models were energy minimized, refined and compared in means of root-mean-square deviation (RMSD) using a derivative of genetic algorithm. Regions altered by mutant V855A in the wild-type HER3 protein are represented as blue bars, colored according to conservation scores.

To elucidate and predict the impact of mutant V855A on the conformation of the wild-type HER3, protein modeling was performed via the automated I-TASSER server [42]. Server predicted models were further refined by submitting the PDB files to Mode Refiner server [43]. Ultimately moderefined predicted models were superimposed on alpha-carbon to unravel structural differences. In addition, we compared moderefined predicted wild-type HER3 with the crystallography obtained model of the HER3 kinase domain (PDB: 3LMG). Although server predicted wild type HER3, differed from the crystallography obtained model, comparison of the predicted wild-type and mutated models showed significant structural differences (data not shown). Superimposition of predicted wild-type / mutated HER3 protein models [44] reveals that the V855A mutation alters the kinase domain and the carboxyl-terminal end of the wild-type HER3 protein as shown by the blue bars along the prediction track depicted in Figure 7.

Taken together, these data suggest that the V855A mutation alters the activity of HER3, which may correlate with a malignant phenotype.

## DISCUSSION

We report a unique case of an adolescent patient with advanced adenocarcinoma of the lung harboring aHER3-V855A kinase mutation in his tumor specimen. Although *HER3* mutations have been identified and characterized in colon and gastric cancers [45], no published studies to our knowledge have reported the occurrence of clinically relevant lung cancer-derived mutations. In addition, we present evidence that the HER3-V855A mutation confers a gain-of-function phenotype and is highly sensitive to HER inhibitors afatinib and pertuzumab. No other examined mutations (*EGFR, HER2, HER4, KRAS* or *BRAF*) were found in the lung tumor specimen of the patient, suggesting that HER3-V855Ais the primary driver for the lung cancer pathogenesis. Our data also strongly suggests that the poor therapeutic response to combination chemotherapy could have been circumvented by molecular targeted therapy. In addition, this mutation is rare as it was found once in a systematic screen of 210 lung cancer specimens phenotypically enriched for adenocarcinoma in patients with non- or limited smoking history.

The V855A mutation targets the activation loop of the *HER3* gene and is homologous to the EGFR-L858R mutation. Amino acid substitutions that have an impact on the biophysical or structural properties of a protein have been described to be pathogenic or deleterious [46]. We demonstrate with protein modeling that the valine to alanine substitution at position 855 has an impact on the kinase domain and carboxyl-terminal end of the wild-type HER3 protein. Based on this *in silico* approach, mutant V855A may thus confer a pathogenic phenotype by destabilizing wild-type HER3.

As demonstrated by prior studies [47] and confirmed by us, the EGFR-L858R mutation is able to transform Ba/F3 cells to IL-3 independent growth and constitutively enhances EGFR autophosphorylation as a homodimer. However, HER3-V855A is unable to transform Ba/F3 cells to either IL-3 independent or ligand-induced growth as a homodimer. Rather, it must associate with its preferred dimerization partner, HER2, upon binding of an exogenous substrate such as NRG1β in order to transform Ba/F3 cells. Multiple lines of evidence support this finding, demonstrating that the wild-type HER3 receptor's impaired kinase activity can be circumvented by ligand-induced heterodimerization; preferably with its HER2 partner [19, 23]. Jaiswal et al. also demonstrated that the major colon/gastric cancer-derived HER3 mutants depend on HER2 for their oncogenic activity, but in contrast to our findings these mutants promote transformation in a ligand-independent manner [45].

The transforming potential of HER3-V855A compared to wild-type HER3 was confirmed by its ability to increase ligand-induced coupling of signal transducers that mediate proliferative and pro-survival responses, indicating a gain-of-function phenotype. Functional data from our cellular models demonstrate that HER3-V855A strongly enhances NRG1β-induced trans-activation of HER2 compared to wild-type HER3. Enhanced trans-activation of HER receptor activity appears to be a central event in receptor tyrosine kinase (RTK) activation by lung-cancer derived EGFR/HER mutations [36]. This activation nonetheless requires a competent HER2 kinase, as our data also reveal that HER3-V855A is unable to induce trans-activation of kinase-dead HER2. This finding thus indicates that HER3 phosphorylation in cells expressing mutant V855A requires the catalytic activity of HER2.

A recent study by Littlefield and colleauges has demonstrated that cancer-associated HER3 variants may not increase the catalytic activity of HER3, but may possibly increase the allosteric activator function and interaction of HER3 to its dimerization partner [48]. Our co-immunoprecipitaton experiments support these findings, where we demonstrate enhanced interaction between the wild-type HER2 protein complex and mutant HER3. This enhanced interaction between the two dimerization partners can consequently lead to increased activation, alterations of the angle of the ATP binding cleft and a more pronounced sensitivity to tyrosine kinase inhibitors [49].

On the other hand, HER3-V855A combined with EGFR does not enhance ligand-induced transformation of Ba/F3 cells. Whereas wild-type HER3 combined with EGFR is able to form colonies under chronic TGFα treatment, the mutant HER3-V855A combination does not. Our findings thus demonstrate that mutant V855A can be delineated into two distinct phenotypes, depending on which HER receptor it associates with. The mechanism, however, behind these differing effects is currently unknown. [48]. Some theoretical possibilities could be due to (1) phosphotransferase activity occurring either through a dissociative (inactivating) or associative (activating) state [50] when mutant V855A is coupled with EGFR or HER2 respectively, or (2) a weaker ligand-induced trans-phosporylation of HER3 by EGFR. In line with this, Shi et al. present data showing that HER3-V855A on its own reduces mant [2′-(3′)-O-(N-methylanthraniloyl)] -ATP binding compared to the wild-type HER3 protein [20]. While the observations of Shi et al. implicate mutant V855A as a suppressor of HER3 kinase activity, further work is needed to establish its capacity to bind ATP when influenced by its relevant HER dimerization partner.

Our analysis also shows that inhibitors targeting the extracellular and kinase domain of the *HER* receptors are effective in suppressing HER3-V855Adriven activity, albeit with differing potency. As expected, afatinib was more effective than erlotinib in suppressing growth and survival signals due to its target specificity and ability to irreversibly bind to the HER kinase. It should be noted, however, that HER3 lacks a conserved cysteine group found in the kinase domain of the other HER family members that is essential for effective binding of afatinib [9]. Afatinib is thus able to exert its potency by inhibiting the trans-activation of HER2 by HER3. Pertuzumab, an inhibitor targeting HER2 dimerization, was also effective in suppressing the transforming potential of mutant V855A; further indicating that coupling with HER2 is essential for its oncogenic activity. Conversely, trastuzumab (an inhibitor targeting the HER2 extracellular domain), is ineffective in blocking mutant V855A activity (unpublished data not included in this report). Previous studies have shown that while pertuzumab efficiently blocks ligand-induced HER3/HER2 heterodimerization [51], trastuzumab is more effective in blocking ligand-independent HER3/HER2 interactions [52]. These findings are consistent with results presented by the research group of Jaiswal in the framework of ligand-independent mutant *HER3* [45]. All tested inhibitors exhibited less potent effects of transformed Ba/F3 cells expressing wild-type HER3 combined with HER2 upon NRG1β stimulation. This provides a strong biological rationale in establishing mutant V855A as a predictive marker in the context of response to HER targeted therapy. Accordingly, our results will also be useful in evaluating targeted treatment approaches in ongoing clinical trials for patients with mutant HER3 NSCLC (CTBE2013000234; HER3-LungNCT02134015).

In conclusion, the identification of HER3-V855A and its functional properties for the first time clearly implicates genomic *HER3* activation in the pathogenesis of lung cancer. *HER3* is traditionally referred to as a kinase-dead or kinase-impaired *HER* family member due to evolutionary divergence at critical residues within its kinase domain [18]. The capacity of a single amino acid substitution in the HER3 kinase to enhance trans-activation of HER2 opens up the possibility that other regulatory mechanisms can be involved in its catalytic function. Our findings thus establish the HER3 kinase as a target for cancer therapy and support its continued assessment and validation as a potential therapeutic modality for personalized-tailored cancer treatment.

## MATERIALS AND METHODS

### Study population

Patients (n= 210) were prospectively enrolled between May 2006 and March 2010 in an academic single-arm multicenter phase II study in Belgium and Luxemburg (FIELT study; NCT00339586). The study was approved by the institutional and ethics review board of each participating center. Histologically or cytologically confirmed locally advanced or metastatic (Stage IIIb or Stage IV) adenocarcinoma of the lung, and a non- to poor smoking history were the key eligibility criteria for the study. Patients with insufficient tumor material for genetic testing or results classified as inconclusive due to a low amount of malignant cells in tumor specimen were excluded from the analysis (n=65). All patients provided separate written informed consent for genetic analysis of their tumor specimens and subsequent inclusion in the treatment phase of the study.

### Mutational screening

Mutation analysis was performed on DNA extracted from three consecutive 10μm thick sections of formalin fixed and paraffin embedded tissue. Tissue sections were examined to verify the presence of a sufficient amount of malignant cells and manually macro-dissected when necessary. Tissue area ranged from 1 mm^2^ to 600 mm^2^, although 40% of the sections were smaller than 5 mm^2^ (mostly needle biopsies). The mutation screening assay at that time (2006) was based on the polymerase chain reaction (PCR)-derived denaturing gradient gel electrophoresis (DGGE) method that covered the genomic regions that encode the tyrosine kinase domain (exons 18-21) of all 4 *HER* family genes. DGGE is a very sensitive method that can detect mutant DNA species based on the differential melting properties of homoduplex and heteroduplex PCR fragments with abnormally migrating bands revealing the presence of a genomic variant [29, 53, 54]. Samples were also analyzed for the presence of somatic mutations of *BRAF* (exons 11 and 15) and *KRAS* (exon 2). Mutations were confirmed by Sanger dideoxy-terminator sequencing according to established methods.

### DNA constructs and site-directed mutagenesis

The pcDNA3.1 HER3 hygro (+) expression plasmid kindly provided by Dr. M. Rødland (*Espen Stang Institute of Pathology, Oslo Norway*) was constructed as previously described [47, 55]. Likewise, the pErbB3-eCFP expression vector was provided by Dr. Rong-Hua Tao (*Okinawa Institute of Science and Technology, Okinawa Japan*) and was constructed as described [56]. To construct pHER2-FLAG-CMV-14, a fragment encoding the full length sequence of HER2 with a *Hind III* site at the N terminus and *Xba I* site at the C terminus was PCR amplified. The resulting PCR fragment was digested with *Hind III* and *Xba I* and subsequently cloned into the p3xFLAG-CMV-14 vector. pcDNA 3.1 neo (+) expressing full length HER2 or HER2-K753M (kinase-dead) was constructed as previously described [57]. pcDNA3.1hygro (+) was purchased from Invitrogen and pcDNA3HER2 neo (+) was obtained from Addgene Inc. The HER3-V855A mutation was generated by the Gene Tailor^TM^ Site-Directed Mutagenesis kit (*Invitrogen*) following the manufacturer's instructions with modifications. Mutagenic oligonucleotides were designed using standard primer design procedures and synthesized by Eurogentec. All mutated constructs were verified by restriction- digestion and sequencing analysis. Primer sequences are available upon request.

### Cell culture

Ba/F3 cells kindly provided by Dr. J. Jiang (*Dana-Farber Cancer Institute*), were maintained in RPMI 1640 (*Gibco, Belgium*) supplemented with 10% fetal bovine serum, 10 units/mL penicillin, 10 μg/mL streptomycin and interleukin-3 (*Sigma*) at 10ng/mL. To generate stable polyclonal cell populations, Ba/F3 cells were electroporated at 220V/1500mF (*SEDD electropore 2000*) with 20μg of a single expression vector or 10μg each for paired combinations. Each of the expression vectors was linearized with restriction endonucleases before transfection. Transfected cells were selected in the presence of 1200μg/mL G418 or 1200 μg/mL hygromycin for single transfections or 1000μg/mL G418 + 1000μg/mL hygromycin for co-transfections. Stable polyclonal cell lines were selected by fluorescence-activated cell sorting analysis using EGFR, HER2 (*BD Biosciences*) and HER3 (*R&D systems*) specific antibodies and cultured for further study. HEK 293T cells (*American Type Culture Collection*) were maintained in DMEM supplemented with 10% fetal bovine serum, 10 units/mL penicillin and 10μg/mL streptomycin. NCI-H292 cells (*American Type Culture Collection*) were maintained in RPMI 1640containing10% fetal bovine serum, 10 units/mL penicillin and 10μg/mL streptomycin. Transient transfection was performed with Lipofectamine^2000^(*Invitrogen*) according to the manufacturer's instructions.

### Inhibitors

The reversible HER-specific TKI erlotinib (Tarceva^®^) and HER2-specific monoclonal antibody, pertuzumab (Perjeta^®^) were provided by Roche. The pan-HER/ErbB TKI afatinib (BIBW 2992) was provided by *Boehringer Ingelheim*. Stock solutions of erlotinib and afatinib were prepared at a concentration of 10 mmol/L in dimethyl sulfoxide (DMSO) and stored at −80°C. The drugs were diluted to a working concentration of 1mmol/L in DMSO before each experiment with a final concentration of 0.1% DMSO used in all experiments.

### Growth assays

Cell growth was assessed by the colorimetric tetrazolium MTS assay (*Promega*). Ba/F3 transfectants, cells were seeded in clear-bottomed 96-well plates at a density of 1×10^4^cells/well in triplicates or quadruplets. Growth factor studies were performed by stimulating cells with 100ng/mL of HER cognate ligands [EGF (*Cell signaling Technology*), TGFα and neuregulin-1β (*Abcam*)]. For drug inhibition studies, the cells were pre-treated with various concentrations of the indicated drugs and subsequently stimulated with 100ng/mL of the indicated HER cognate ligands. After 7 days, the numbers of viable cells were analyzed at an absorbance of 490nm using a96-well microplate reader (*Labsystems*) according to the manufacturer's instructions. All drug treatment conditions were compared to the vehicle control. IC_50_ values were assessed by the Biosoft^®^ CalcuSyn version 2.0 software (*Biosoft*).

### HER tyrosine phosphorylation, immunoprecipitation, and Western blot

To analyze tyrosine phosphorylationof HER proteins, Ba/F3 transfectants were incubated at 37°C for 16 hours in serum free conditions to obtain basal tyrosine phosphorylation. Aliquots containing 4×10^6^ cells were stimulated with their indicated HER cognate ligand for 10 min and lysed in a Tris-buffer [25 mmol/L Tris-HCL (pH 7.4), 150 mmol/L NaCl, 1% Triton-x, 5ug/mL leupeptin]containing a protease and phosphatase inhibitor cocktail (*Sigma*). Lysates were cleared by centrifugation and protein concentration was determined by the Bradford protein assay kit (*Bio-Rad*) and equivalent amount of protein were loaded on a 7.5% resolving acrylamide gel and blotted on a polyvinyliden fluoride membrane (PVDF). The membrane was then subjected to an immunodetection procedure using the indicated antibodies:phospho-HER2 (Tyr 1248), phospho-HER3 (Tyr 1289), HER2, HER3 and AKT/PKB from Cell Signaling, phospho-AKT/PKB (Ser 473) from Invitrogen, and β-actin from Sigma-Aldrich. Horseradish peroxidase (HRP)-conjugated secondary antibodies (*GE Healthcare; Cell Signaling*) and a chemoluminescent detection kit (*Perkin-Elmer*) were used to detect the indicated proteins. To assess the inhibition of HER tyrosine phosphorylation and their downstream targets, Ba/F3 transfectants were incubated at 37°C for 16 hours in serum free conditions. The cells were then incubated for 2 hours at 37°C with required inhibitors followed by10 minute stimulation with NRG1β. Cells were subsequently collected and used for Western blot analysis as described. To assess the protein-protein interaction between HER3 and HER2, Ba/F3 transfectants were stimulated for 10 minutes with or without 100ng/ml NRG1β after one hour incubation in serum free conditions and precipitated with the 5μg of the indicated antibodies and 75μl of G Sepharose beads (*GE healthcare*). The precipitates were eluted with SDS sample buffer /DTT and subjected to Western blot analysis as described.

### Colony formation assay

Stable Ba/F3 transfectants (1×10^5^) were suspended in 0.5% methylcellulose solution (*Sigma*) containing the required stimulants or inhibitors. Cell suspensions were plated on to 6 well plates, incubated for 21 days and assessed for colony formation. The mean colony size for each condition was determined by means of computerized Photoshop CS6 analysis.

### Annexin V/7-AAD assay

Cellular apoptosis was determined by the Annexin V-APC and 7-Amino-actinomycin D (7-AAD) binding assay (BD Biosciences). Briefly, inhibitor treated (erlotinib (1μM), afatinib (1μM) and pertuzumab (20 μg/mL)) Ba/F3 co-transfectants were seeded at a concentration of approximately 1 × 10^6^ cells/mL and collected after 5 days. The cells were stained according to the manufactures instructions and analyzed by fluorescence-activated cell sorting (*Becton Dickinson*). The percentage of early and late apoptotic cells were calculated using FlowJo version 7.5.2 software (*TreeStar*).

### Protein crystallography

The X-ray crystallographic structure representing the inactive state of the HER3 kinase domain (PDB: 3LMG) was analyzed using Deepview-Swiss-PDB viewer v4.1.0 [58] and POV-RAY v3.6 [59] bioinformatics tools.

### Protein modeling and prediction

Predicted models were generated by submitting raw wild-type (Protein: NP_001973.2, cdna: NM_001982.3) and mutated HER3 p.V855A fasta sequences to the automated I-TASSER server [42]. Generated models were then energy minimized via two cycles of steepest descent consisting of 50 steps each and one cycle of conjugate gradient consisting of 200 steps with a minimum ΔE of 0.01kJ/mol together with a harmonic constraint of 100 kJ/mol. Models were also refined using Modrefiner [43]. Models were compared to each other and to crystal structures of HER3 kinase domain (PDB: 3LMG) and extracellular domain (PDB: 1M6B) by X-ray diffraction. Server predicted models were compared to each other by RMSD difference. Conservation data were generated by alignment between *Homo sapiens*, *Mus musculus*, *Rattus norvegicus and Pongo abelii*. All renderings and modifications were performed using bioinformatics tools as described [58, 59].

### Statistical Analysis

Results are representative of three independent experiments unless stated otherwise. Values are presented as the mean ± standard error of mean (SEM). The unpaired two-tailed t-test was utilized to compare the means of two groups while the Mann Whitney *U* test was utilized when comparing colony size in the colony formation assay. Statistical significance is reported as follows:*, P < 0.05, **, P < 0.01 and ***, P < 0.001.

## SUPPLEMENTARY MATERIAL FIGURES


