# Supplementary material for: Identification of a novel HER3 activating mutation homologous to EGFR-L858R in lung cancer
Source: Oncotarget. 2015 Dec 9;7(3):3068–83. doi: 10.18632/oncotarget.6585 (PMC4823091; doi:10.18632/oncotarget.6585)
Supplement: Supplementary file 1 [file oncotarget-07-3068-s001.pdf]

Identification of a novel HER3 activating mutation homologous to EGFR-L858R in lung cancer

Supplementary Material

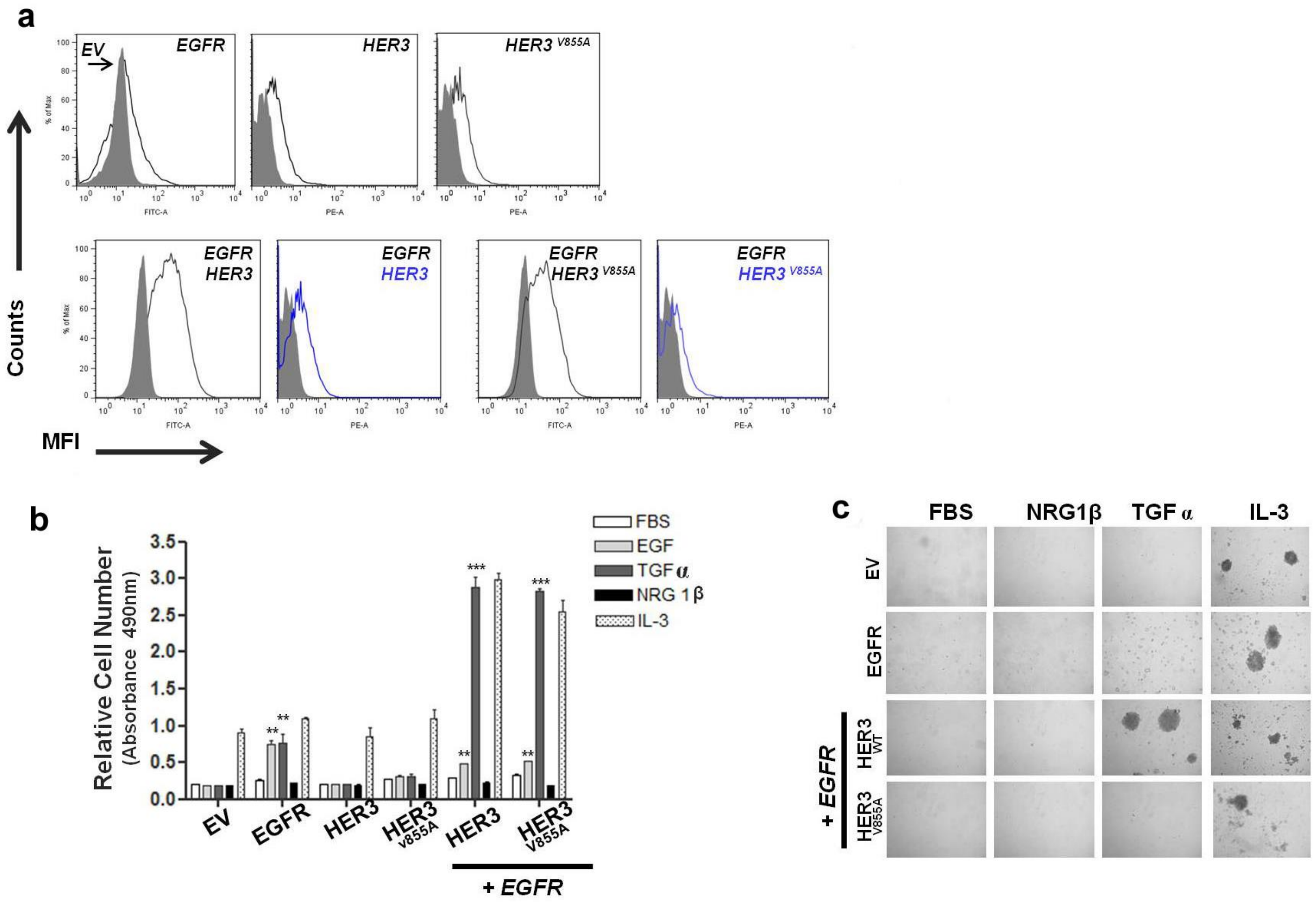

Supplemental Figure 1: related to Figure 3. HER3-V855A combined with EGFR does not enhance ligand-induced transformation of Ba/F3 cells.

(a) Selected Ba/F3 transfectants were analyzed for cell surface protein expression by staining with HER-specific antibodies to confirm recombinant protein expression. EGFR and HER3 co-transfectants were labeled with FITC-conjugated anti-EGFR or PE-conjugated anti-HER3 antibodies. (b) Ba/F3 transfectants were cultured for 7 days in the absence or presence of the indicated ligands. Cell growth was analyzed by the MTS assay. (c) Ba/F3 co-transfectants were subjected to a methyl cellulose based colony formation assay in the presence of the indicated ligands for 21 days. Magnification, 20X.

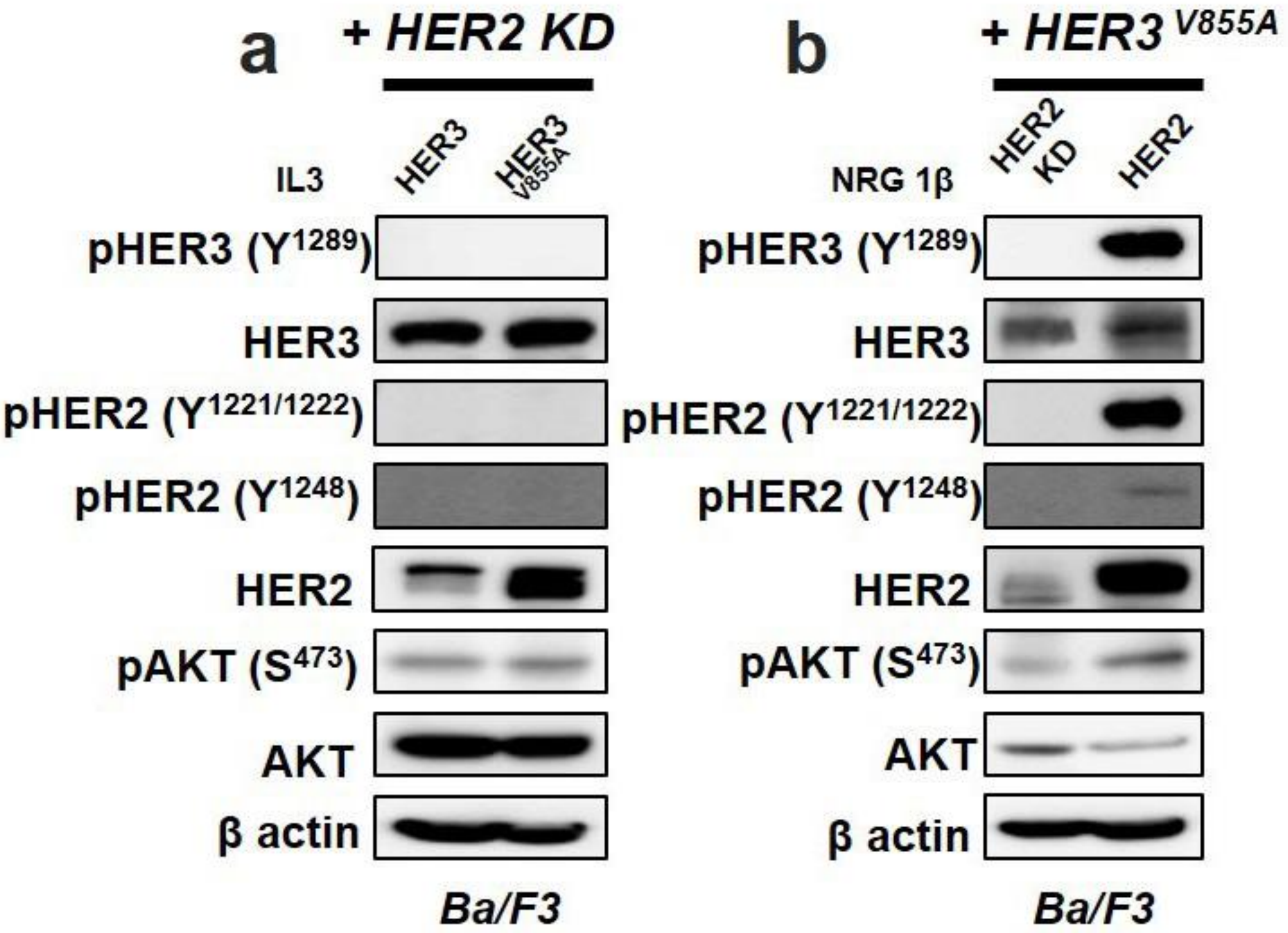

Supplemental Figure 2: related to Figure 3. Effect of chronic neuregulin1β treatment on HER phosphorylation in Ba/F3 cells co-expressing HER3-V855A and wild-type or kinase-dead (KD) forms of HER2.

(a,b) Ba/F3 co-transfectants were cultured for 3 -5 days in growth medium containing IL-3 or NRG1β. Cell lysates were collected for immunoblot analysis with the indicated antibodies.

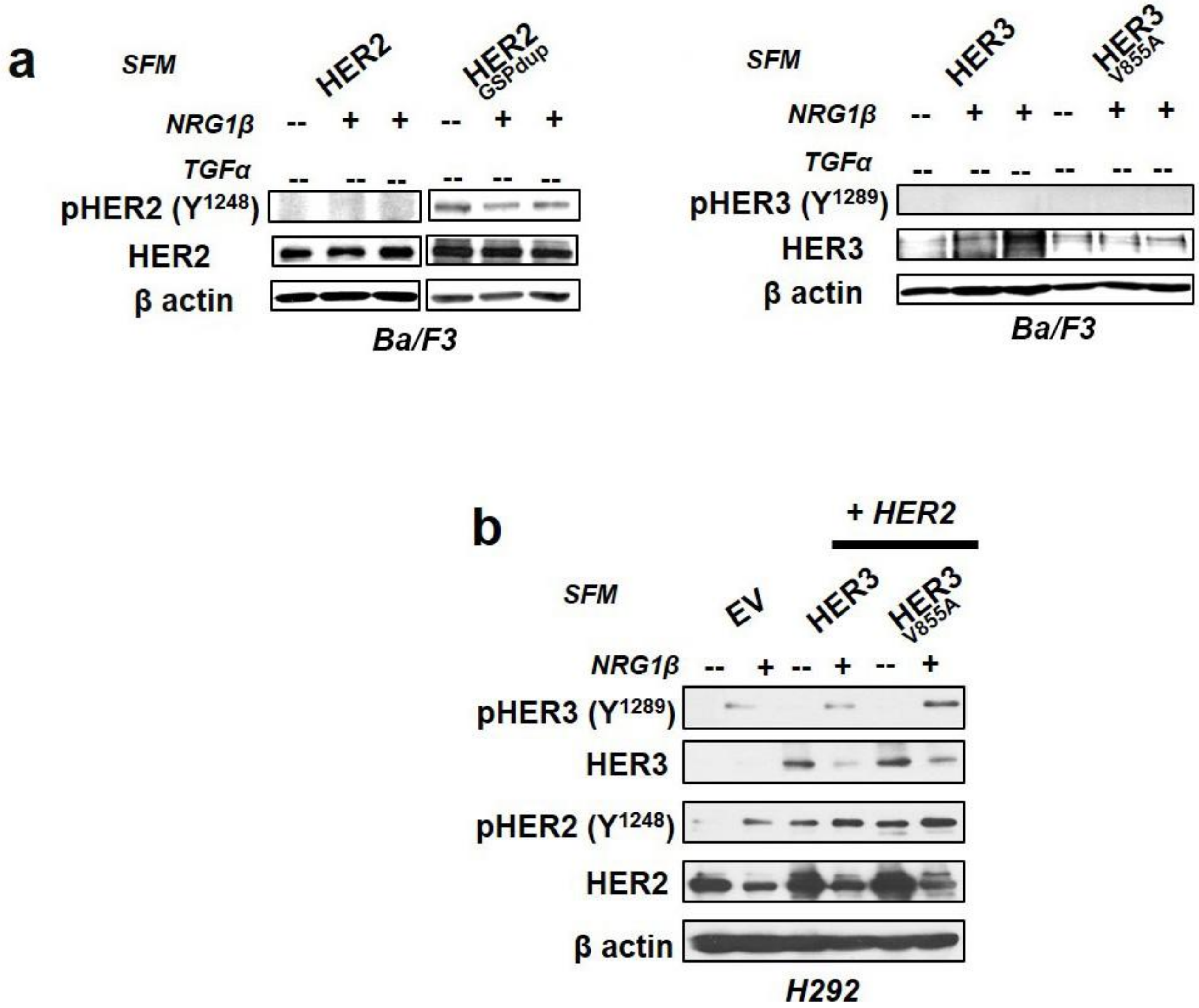

Supplemental Figure 3: related to Figure 4. Effect of acute neuregulin1β treatment in Ba/F3 and H292 (NSCLC cells) transfectants.

(a,b)Western blot analysis of Ba/F3 and H292 transfectants treated for 16hrs in serum free conditions (SFM) and stimulated for 10 minutes withNRG1β. The cells were subsequently lysed and subjected to immunoblot analyses.HER2-GSP 781_783dup (HER2-GSPdup) is a NSCLC-derived HER2 variant.
